# Supplementary material for: Clusters of microRNAs emerge by new hairpins in existing transcripts
Source: Nucleic Acids Res. 2013 Jun 17;41(16):7745–52. doi: 10.1093/nar/gkt534 (PMC3763532; doi:10.1093/nar/gkt534)
Supplement: Supplementary Data [file supp_41_16_7745__index.html]

Clusters of microRNAs emerge by new hairpins in existing transcripts — Clusters of microRNAs emerge by new hairpins in existing transcripts — Supplementary Data 

# Clusters of microRNAs emerge by new hairpins in existing transcripts

## 

files

**Files in this Data Supplement:**

- Supplementary Data - zip file
