# Supplementary material for: Clusters of microRNAs emerge by new hairpins in existing transcripts
Source: Nucleic Acids Res. 2013 Jun 17;41(16):7745–52. doi: 10.1093/nar/gkt534 (PMC3763532; doi:10.1093/nar/gkt534)
Supplement: Supplementary Data [file supp_gkt534_nar-01030-z-2013-File005.zip › NAR-01030-2013 Suppl Files/Supplementary_Dataset_1.pdf]

Dataset S1. Evolutionary history of *Drosophila melanogaster* microRNA clusters

mir-100/let-7/mir-125

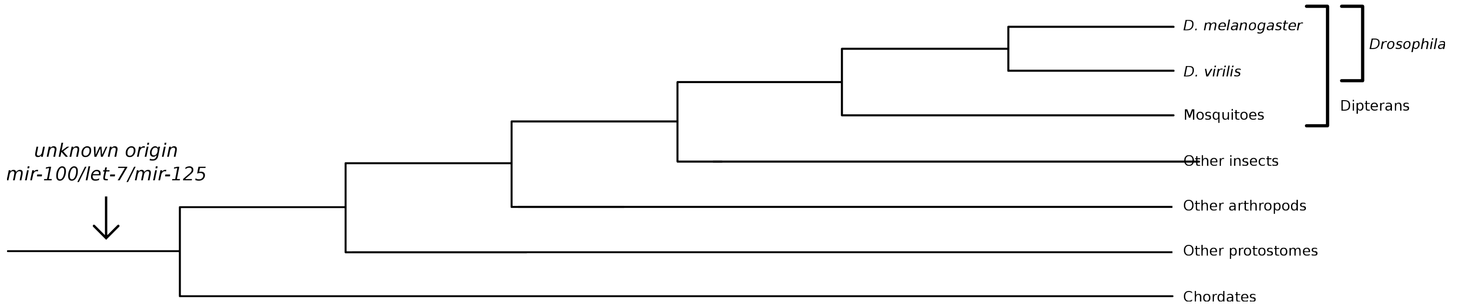

mir-999/mir-4969

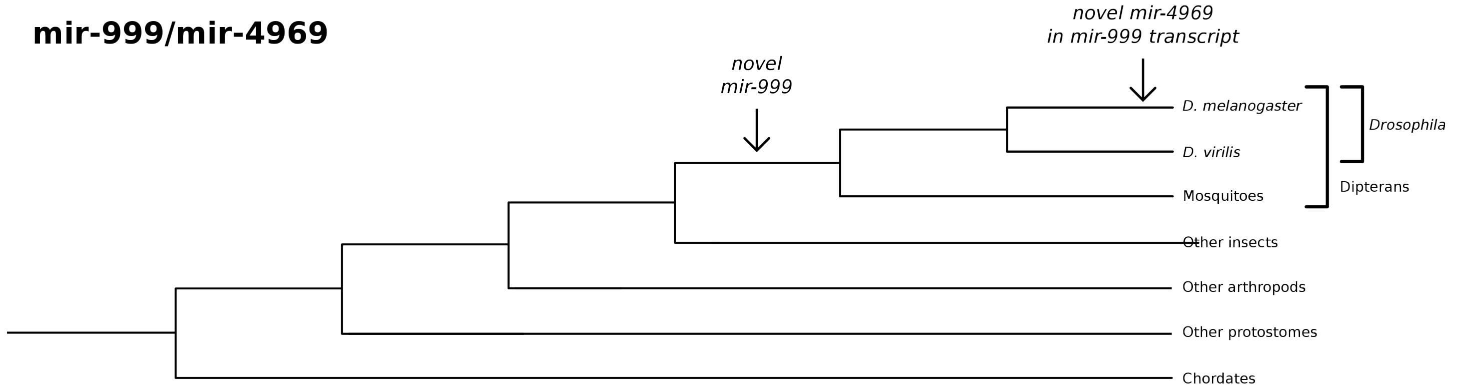

mir-982/mir-303/mir-983-1/mir-983-2/mir-984

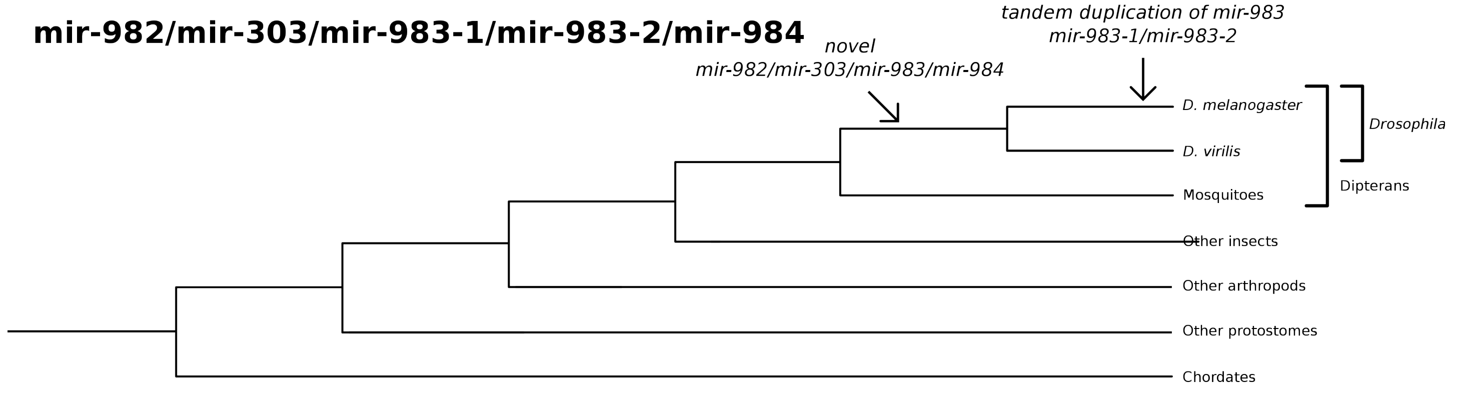

mir-969/mir-210

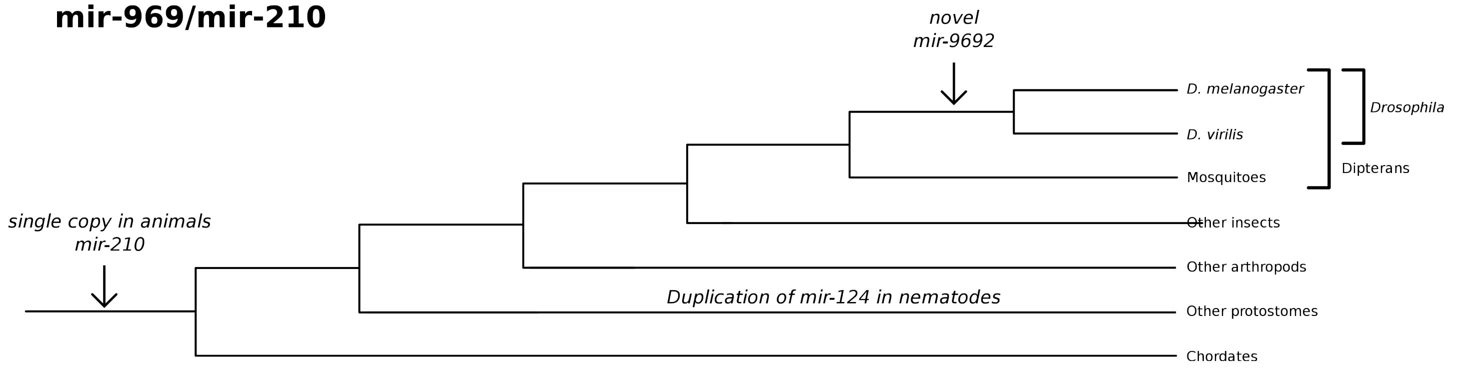

mir-124/mir-287

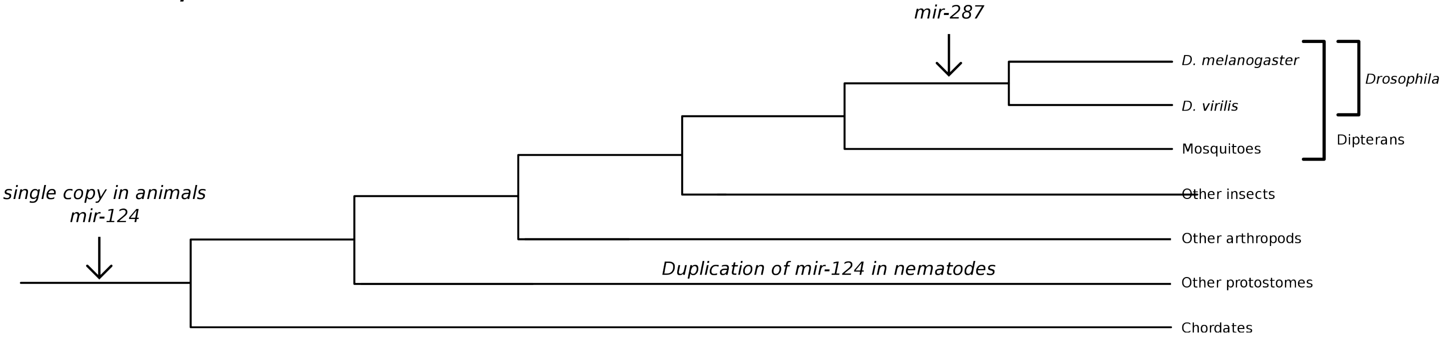

mir-972/mir-973/mir-974/mir-2499/mir-4966/mir-975/mir-976 /mir-977/mir-978/mir-979

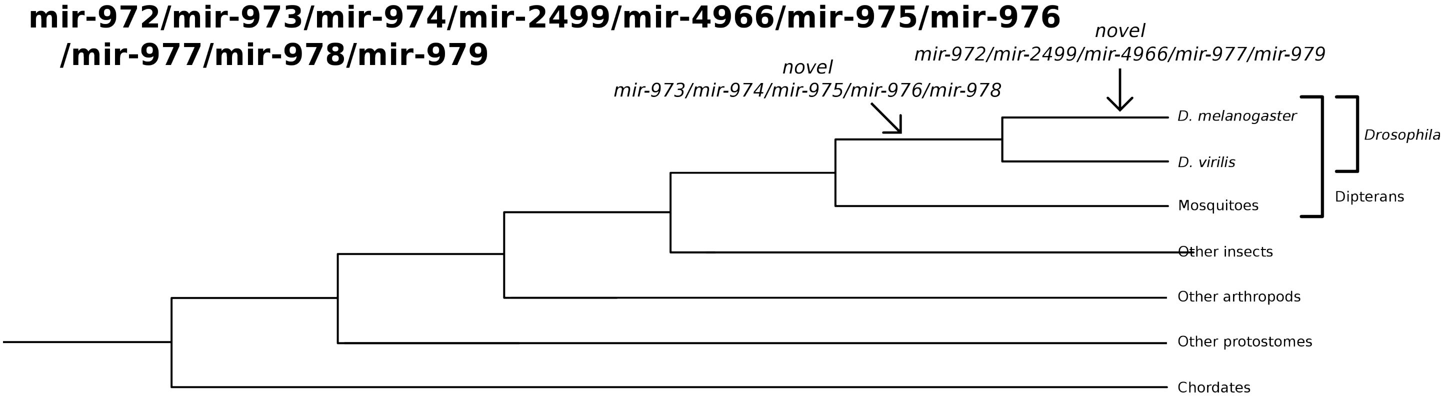

mir-994/mir-318

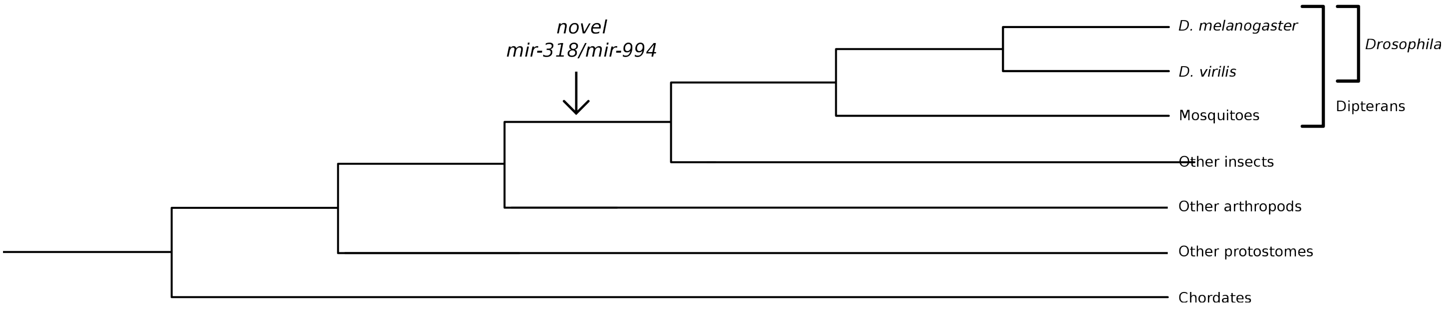

mir-310/mir-311/mir-312/mir-313/mir-2498/mir-991/mir-992

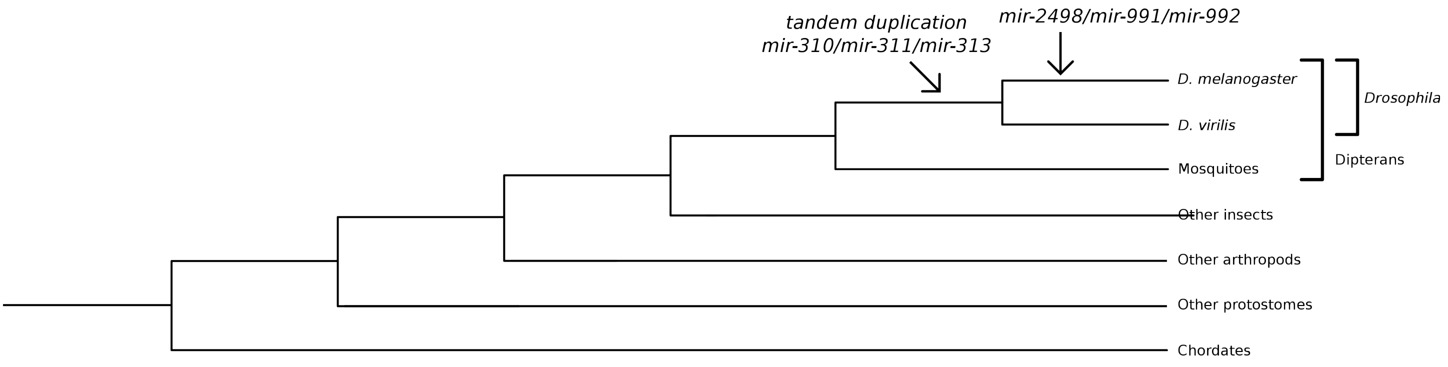

mir-6-3/mir-6-2/mir-6-1/mir-5/mir-4/mir-286/mir-3/mir-309

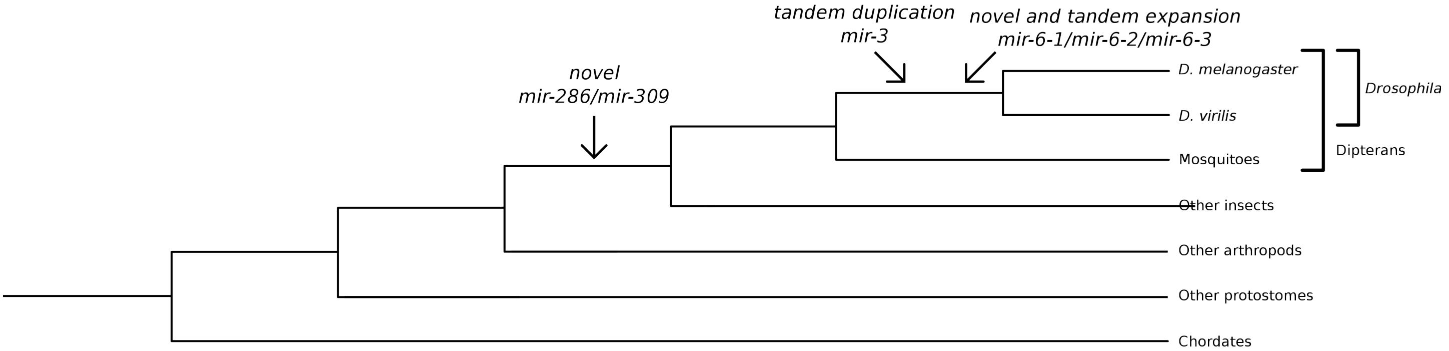

mir-9c/mir-306/mir-79/mir-9b

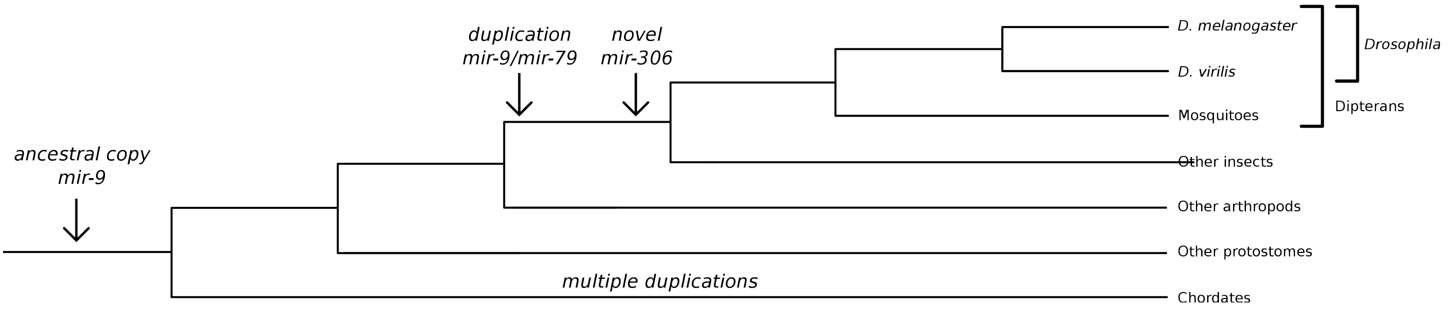

mir-283/mir-304/mir-12

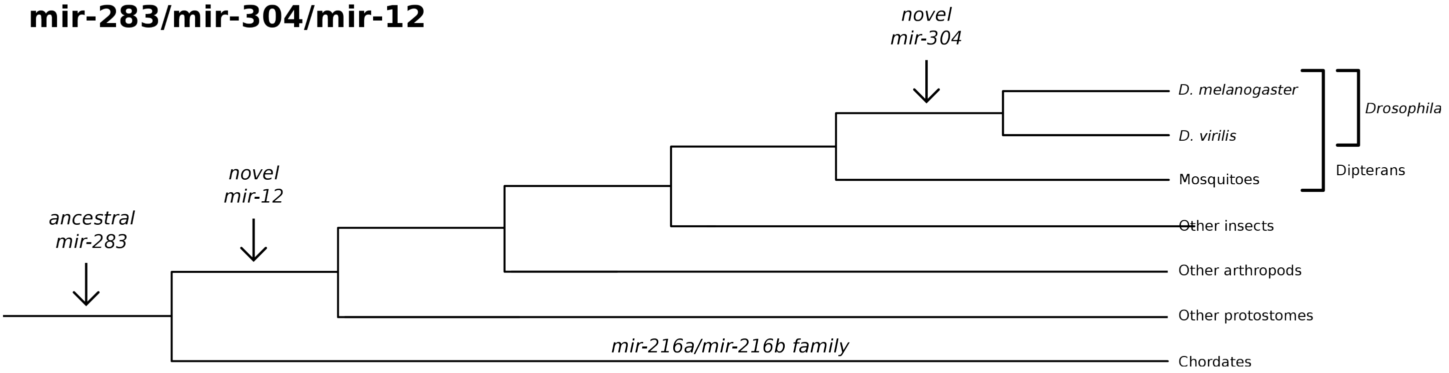

mir-275/mir-305

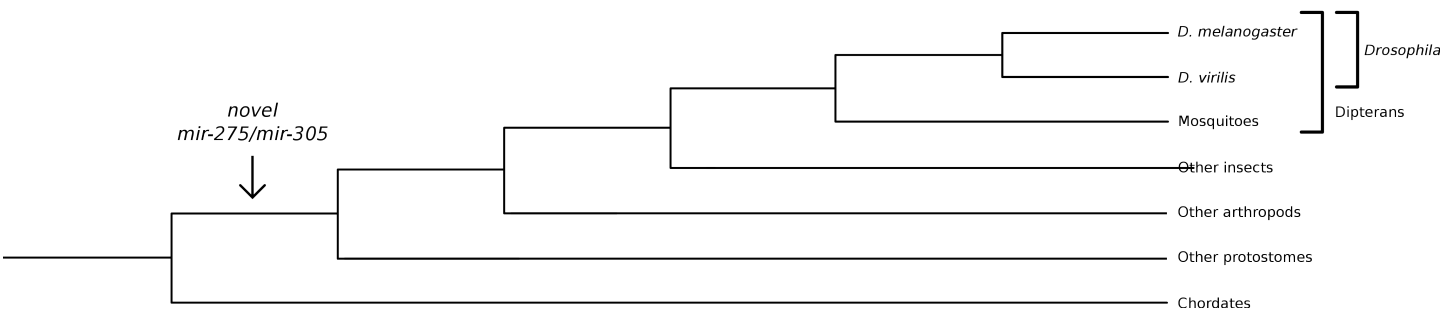

mir-959/mir-960/mir-961/mir-962/mir-963/mir-964

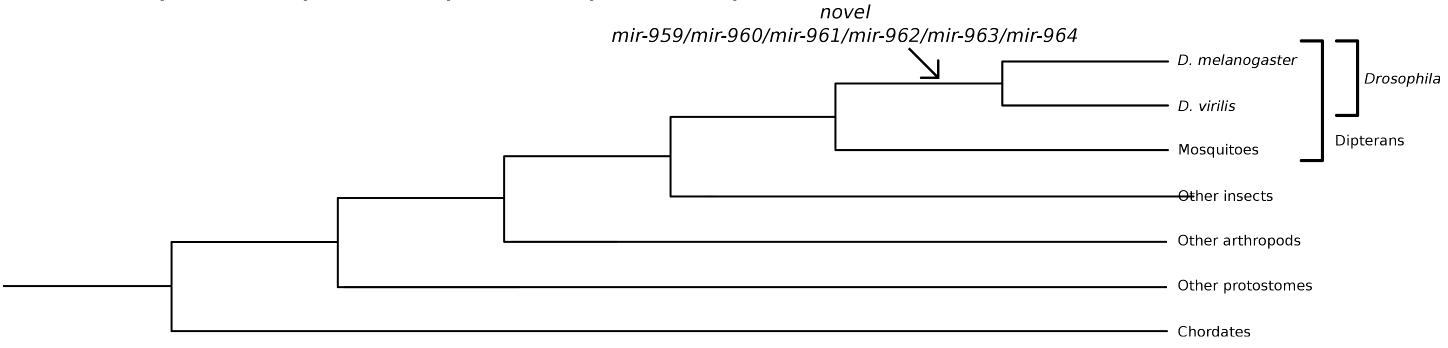

mir-1002/mir-968

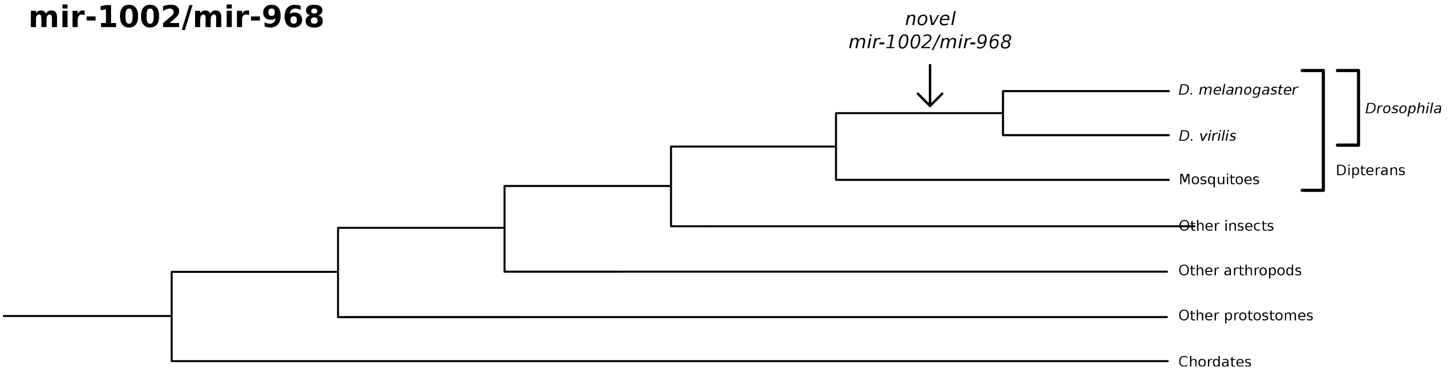

mir-281-1/mir-281-2

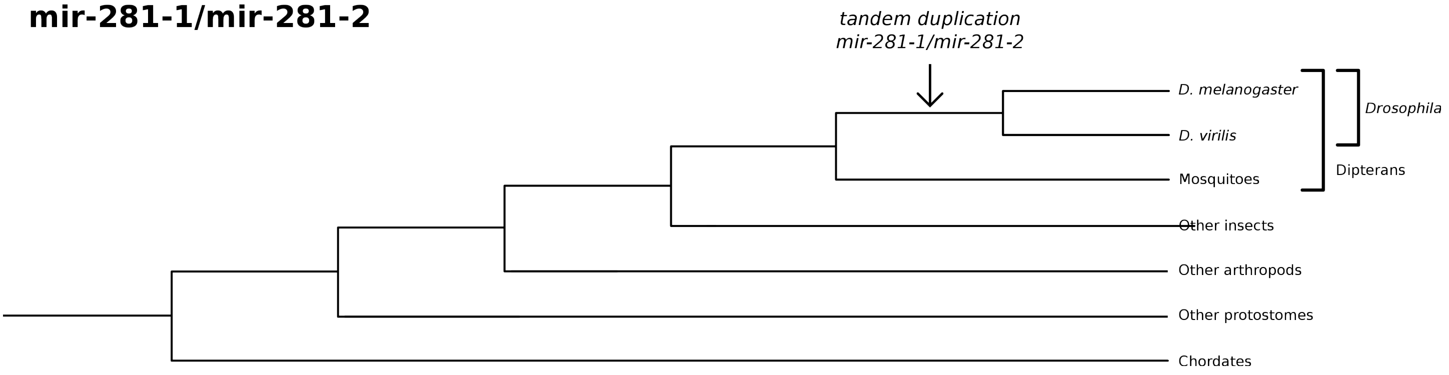

mir-279/mir-996

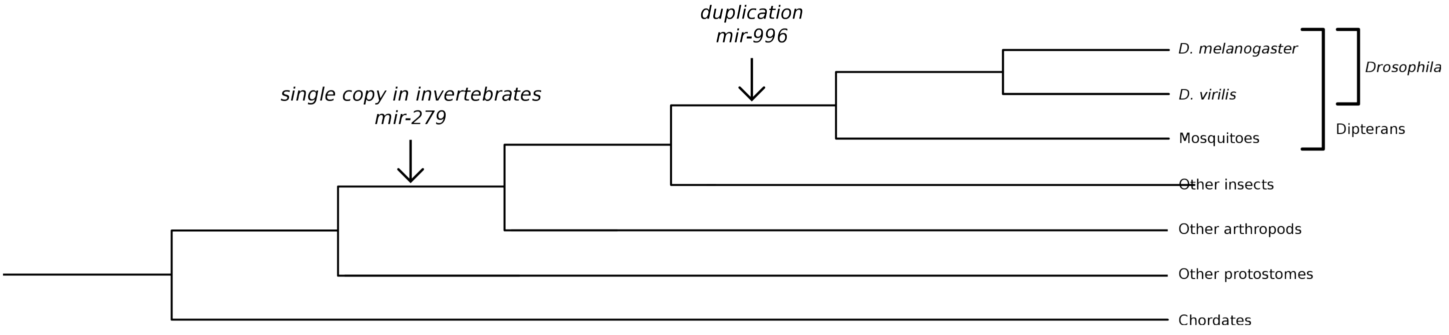

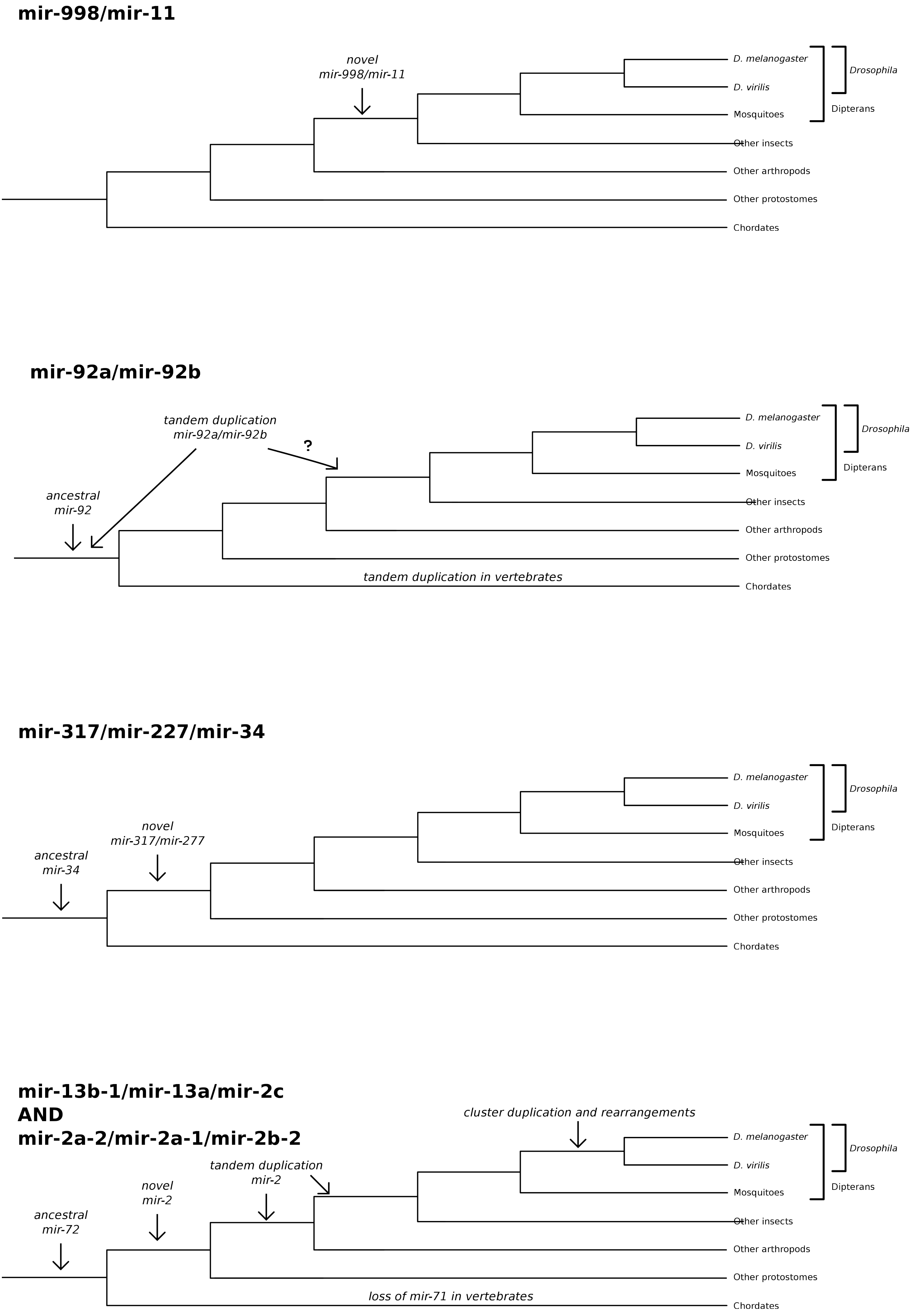

mir-998/mir-11
novel
mir-998/mir-11
D. melanogaster
D. virilis
Drosophila
Mosquitoes
Dipterans
Other insects
Other arthropods
Other protostomes
Chordates
mir-92a/mir-92b
tandem duplication
mir-92a/mir-92b
?
ancestral
mir-92
D. melanogaster
D. virilis
Drosophila
Mosquitoes
Dipterans
Other insects
Other arthropods
Other protostomes
tandem duplication in vertebrates
Chordates
mir-317/mir-227/mir-34
ancestral
mir-34
novel
mir-317/mir-277
D. melanogaster
D. virilis
Drosophila
Mosquitoes
Dipterans
Other insects
Other arthropods
Other protostomes
Chordates
mir-13b-1/mir-13a/mir-2c
AND
mir-2a-2/mir-2a-1/mir-2b-2
cluster duplication and rearrangements
ancestral
mir-72
novel
mir-2
tandem duplication
mir-2
D. melanogaster
D. virilis
Drosophila
Mosquitoes
Dipterans
Other insects
Other arthropods
Other protostomes
loss of mir-71 in vertebrates
Chordates
