# Supplementary material for: Clusters of microRNAs emerge by new hairpins in existing transcripts
Source: Nucleic Acids Res. 2013 Jun 17;41(16):7745–52. doi: 10.1093/nar/gkt534 (PMC3763532; doi:10.1093/nar/gkt534)
Supplement: Supplementary Data [file supp_gkt534_nar-01030-z-2013-File005.zip › NAR-01030-2013 Suppl Files/Supplementary_Figure_1.pdf]

Figure S1. Phylogenetic tree of the mir-92/mir-25 microRNA family. Sequences were downloaded from miRBase (1) and aligned with ClustalX 2.1 (2). Only mature sequences were use to build the phylogenetic tree with ClustalX: neighbour joining with Kimura correction and 1,000 bootstrap replicates.

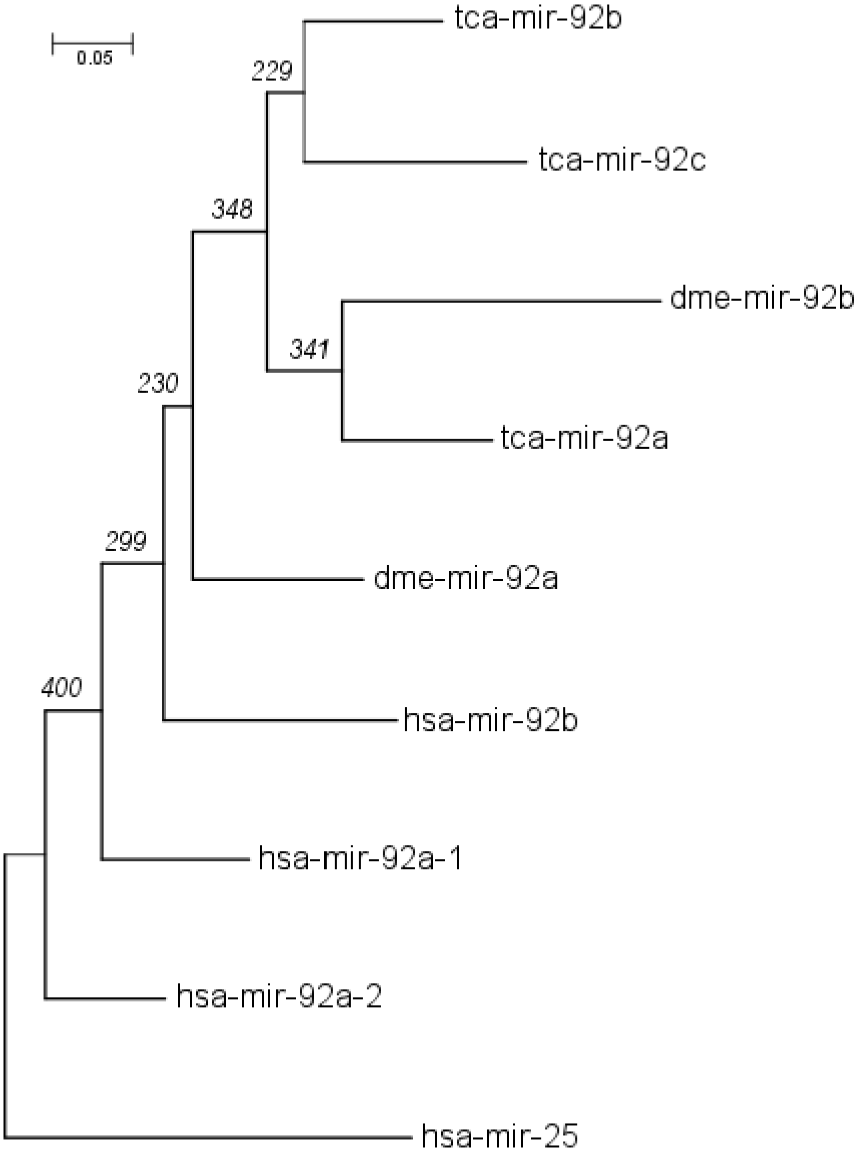

References

1. Kozomara A, Griffiths-Jones S (2011) miRBase: integrating microRNA annotation and deep-sequencing data. *Nucleic Acids Res* 39:D152–157.

2. Larkin MA et al. (2007) Clustal W and Clustal X version 2.0. *Bioinformatics* 23:2947–2948.
