# Supplementary material for: Clusters of microRNAs emerge by new hairpins in existing transcripts
Source: Nucleic Acids Res. 2013 Jun 17;41(16):7745–52. doi: 10.1093/nar/gkt534 (PMC3763532; doi:10.1093/nar/gkt534)
Supplement: Supplementary Data [file supp_gkt534_nar-01030-z-2013-File005.zip › NAR-01030-2013 Suppl Files/Supplementary_Table_1.pdf]

Table S1. Genome assemblies used in this work

Species	Version	Source
Acyrthosiphon pisum	1.0	http://www.ncbi.nih.gov/genomes
Aedes aegypti	AaegL1	http://www.vectorbase.org
Anopheles gambiae	2.1	http://www.ncbi.nih.gov/genomes
Apis mellifera	4.0	http://www.ncbi.nih.gov/genomes
Bombyx mori	2.0	http://www.ncbi.nih.gov/genomes
Branchiostoma floridae	2.0	http://genome.jgi-psf.org
Caenorhabditis elegans	7.1	http://www.wormbase.org
Capitella teleta	1.0	http://genome.jgi-psf.org
Ciona intestinalis	1.0	http://www.ncbi.nih.gov/genomes
Culex quinquefasciatus	CpipJ1	http://www.vectorbase.org
Daphnia pulex	2006.09	http://wfleabase.org
Drosophila ananassae	1.0	http://rana.lbl.gov/drosophila
Drosophila melanogaster	5.0	http://flybase.org
Drosophila pseudoobscura	2.0	http://flybase.org
Drosophila sechelia	1.0	http://www.broadinstitute.org
Drosophila virilis	1.0	http://rana.lbl.gov/drosophila
Drosophila willistoni	1.0	http://rana.lbl.gov/drosophila
Drosophila yakuba	2.1	http://rana.lbl.gov/drosophila
Gallus gallus	2.1	http://www.ncbi.nih.gov/genomes
Homo sapiens	37.1	http://www.ncbi.nih.gov/genomes
Lottia gigantea	1.0	ftp://ftp.jgi-psf.org/pub/JGI_data/Lottia_gigantea
Schistosoma japonicum	2.0	http://www.chgc.sh.cn/japonicum/
Schistosoma mansoni	5.1	http://www.sanger.ac.uk/research/areas/pathogengenetics
Tribolium castaneum	3.0	http://www.ncbi.nih.gov/genomes
