# Supplementary material for: Clusters of microRNAs emerge by new hairpins in existing transcripts
Source: Nucleic Acids Res. 2013 Jun 17;41(16):7745–52. doi: 10.1093/nar/gkt534 (PMC3763532; doi:10.1093/nar/gkt534)
Supplement: Supplementary Data [file supp_gkt534_nar-01030-z-2013-File005.zip › NAR-01030-2013 Suppl Files/Supplementary_Table_2.pdf]

Table S2. MicroRNA expression datasets

GEO number	Tissue / stage	Reference
GSM240749	head	Chung et al. *Curr Biol* 2008, 18:795
GSM272651	S2 cell	Chung et al. *Curr Biol* 2008, 18:795
GSM272652	S2 cell	Chung et al. *Curr Biol* 2008, 18:795
GSM272653	Kc cell	Chung et al. *Curr Biol* 2008, 18:795
GSM275691	imaginal disc	Chung et al. *Curr Biol* 2008, 18:795
GSM286601	head	Chung et al. *Curr Biol* 2008, 18:795
GSM286602	whole body	Chung et al. *Curr Biol* 2008, 18:795
GSM286603	whole body	Chung et al. *Curr Biol* 2008, 18:795
GSM286604	embryo	Chung et al. *Curr Biol* 2008, 18:795
GSM286605	embryo	Chung et al. *Curr Biol* 2008, 18:795
GSM286606	embryo	Chung et al. *Curr Biol* 2008, 18:795
GSM286607	embryo	Chung et al. *Curr Biol* 2008, 18:795
GSM286611	embryo	Chung et al. *Curr Biol* 2008, 18:795
GSM286613	embryo	Chung et al. *Curr Biol* 2008, 18:795
GSM322208	3rd instar larvae	
GSM322219	2-4 day old pupae	
GSM322245	3rd instar larvae	
GSM322338	2-4 day old pupae	
GSM322533	adult female head	
GSM322543	adult male head	
GSM360256	1st instar larvae	
GSM360257	1st instar larvae	
GSM360260	0-1 day old pupae	
GSM360262	0-2 day old pupae	
GSM364902	12-24hr embryo	
GSM280082	from 2-4 day old flies	
GSM280083	from 2-4 day old flies	
GSM280084	from 2-4 day old flies	
GSM280085	from 2-4 day old flies	
GSM280086	from 2-4 day old flies	
GSM280087		
GSM280088		
GSM399105	imaginal disc/brain	
GSM399106	female body	
GSM399107	male body	
GSM371638		
GSM180328	adult heads	Ruby et al. *Genome Res* 2007, 17:1850

GSM180329	adult bodies	Ruby et al. *Genome Res* 2007, 17:1850
GSM180330	very early embryo (0-1)	Ruby et al. *Genome Res* 2007, 17:1850
GSM180331	early embryo (2-6)	Ruby et al. *Genome Res* 2007, 17:1850
GSM180332	mid embryo (6-10)	Ruby et al. *Genome Res* 2007, 17:1850
GSM180333	late embryo (12-24)	Ruby et al. *Genome Res* 2007, 17:1850
GSM180334	larvae: 1st instar and 3rd instars	Ruby et al. *Genome Res* 2007, 17:1850
GSM180335		Ruby et al. *Genome Res* 2007, 17:1850
GSM180336	pupae: 0-1 day, 0-2 day, 2-4 day	Ruby et al. *Genome Res* 2007, 17:1850
GSM180337	tissue culture cells	Ruby et al. *Genome Res* 2007, 17:1850
